# Disclosure of Genetic Information and Change in Dietary Intake: A Randomized Controlled Trial

**DOI:** 10.1371/journal.pone.0112665

**Published:** 2014-11-14

**Authors:** Daiva E. Nielsen, Ahmed El-Sohemy

**Affiliations:** Department of Nutritional Sciences, University of Toronto, 150 College St, Toronto, ON, M5S 3E2, Canada; University of Utah, United States of America

## Abstract

**Background:**

Proponents of consumer genetic tests claim that the information can positively impact health behaviors and aid in chronic disease prevention. However, the effects of disclosing genetic information on dietary intake behavior are not clear.

**Methods:**

A double-blinded, parallel group, 2∶1 online randomized controlled trial was conducted to determine the short- and long-term effects of disclosing nutrition-related genetic information for personalized nutrition on dietary intakes of caffeine, vitamin C, added sugars, and sodium. Participants were healthy men and women aged 20–35 years (n = 138). The intervention group (n = 92) received personalized DNA-based dietary advice for 12-months and the control group (n = 46) received general dietary recommendations with no genetic information for 12-months. Food frequency questionnaires were collected at baseline and 3- and 12-months after the intervention to assess dietary intakes. General linear models were used to compare changes in intakes between those receiving general dietary advice and those receiving DNA-based dietary advice.

**Results:**

Compared to the control group, no significant changes to dietary intakes of the nutrients were observed at 3-months. At 12-months, participants in the intervention group who possessed a risk version of the *ACE* gene, and were advised to limit their sodium intake, significantly reduced their sodium intake (mg/day) compared to the control group (−287.3±114.1 vs. 129.8±118.2, p = 0.008). Those who had the non-risk version of *ACE* did not significantly change their sodium intake compared to the control group (12-months: −244.2±150.2, p = 0.11). Among those with the risk version of the *ACE* gene, the proportion who met the targeted recommendation of 1500 mg/day increased from 19% at baseline to 34% after 12 months (p = 0.06).

**Conclusions:**

These findings demonstrate that disclosing genetic information for personalized nutrition results in greater changes in intake for some dietary components compared to general population-based dietary advice.

**Trial Registration:**

ClinicalTrials.gov NCT01353014

## Introduction

Personal genetic information has become easily obtainable, in large part due to the advancement of the consumer genetic testing industry. As a result of the decreasing costs to carry out genotyping, individuals can now receive personalized feedback regarding their susceptibility to a number of different health conditions at a relatively low cost [1]. The impact that this information may have on health behaviors is of particular interest [2], [3], since chronic diseases such as cardiovascular disease and type 2 diabetes have become major public health concerns. There is considerable evidence that these conditions are associated with a number of modifiable health behaviors such as diet, physical activity and smoking, but lifestyle interventions aimed at achieving positive health behavior changes are often ineffective at producing the long-term changes necessary to mitigate disease risk [4]. As a result, proponents of personalized medicine claim that health recommendations tailored to an individual's genetic profile may be more effective at producing behavior change than generic population-based recommendations. A growing body of qualitative research shows strong public interest in genomics and personalized medicine for disease prevention [5]–[9], but there is limited quantitative evidence to support the claim that personalized genomics can be employed as a useful prevention tool.

The study of how human genetic variations modify an individual's response to diet on various health outcomes, often referred to as nutrigenomics or nutrigenetics, is a key part of personalized medicine [10] because nutrition is arguably one of the most important modifiers of chronic disease risk [11]. Many direct-to-consumer genetic tests provide single nucleotide polymorphism (SNP)-based estimates of disease susceptibility that do not take into account environmental factors. For complex diseases, including diet-related chronic diseases, risk estimates based solely on genetic variation without consideration of environmental interactions can be inaccurate [12]. Therefore, genetic testing for personalized nutrition using *modifier* or *metabolic* genes may have the potential to be more useful than genetic testing for disease risk using disease susceptibility genes because the advice that is given from a personalized nutrition test is more specific and actionable than advice from a disease susceptibility test. Indeed, a previous study demonstrated that individuals consider DNA-based dietary advice to be more useful and understandable than general population-based dietary recommendations, and individuals report that they would be more motivated to change their diet if provided with personalized nutrition information based on their genetics [13]. Individuals who have had their genomes analyzed report that the genetic information impacted their dietary behaviors, although the genetic information they received was not necessarily linked to any specific dietary modification [14], [15]. Despite this evidence, no previous study has examined the effect of disclosing personalized genetic information based on nutrigenomics testing on dietary intake behavior. In addition, previous studies investigating the impact of personal genomic information related to disease susceptibility on health behaviors have lacked long-term follow-up data. As a result, the short- and long-term effects of personal genomic information on health behaviors are largely unknown. Therefore, the objective of the present study was to determine the short- and long-term effects of disclosing genetic information for personalized nutrition on dietary intake in a population of young adults using a randomized controlled trial (Table 1).

**Table 1:** Prevalence of risk alleles in intervention group (n = 92) and associated risk.

Dietary Component	Gene	Risk Allele Non-Risk Allele		Associated Risk
		n (%)		
Caffeine	*CYP1A2*	48 (52)	44 (48)	Increased risk of myocardial infarction and hypertension when consuming above 200 mg of caffeine/day
				*General recommendation:* ≤300 mg/day for women of child-bearing age
				≤400 mg/day for other adults
				*Targeted Recommendation:* ≤200 mg/day for those with risk version of *CYP1A2*
Vitamin C	*GSTM1* + *GSTT1*	52 (57)	40 (43)	Increased risk of serum ascorbic acid deficiency when consuming below the RDA for vitamin C
				*General recommendation:* RDA^a^ for women: ≥75 mg/day
				RDA for men: ≥90 mg/day
				*Targeted Recommendation:* Same as general recommendation
Added Sugars	*TAS1R2*	41 (45)	51 (55)	Increased risk of over-consuming sugars
				*General recommendation:* ≤10% energy/day
				*Targeted Recommendation:* Same as general recommendation
Sodium	*ACE*	64 (70)	28 (30)	Increased risk of sodium-sensitive hypertension when consuming above the AI for sodium
				*General recommendation:* UL^b^: ≤2300 mg/day
				*Targeted Recommendation:* AI^c^: ≤1500 mg/day for those with risk version of *ACE*

a RDA: Recommended dietary allowance.

b UL: Tolerable upper intake level.

c AI: Adequate intake.

## Materials and Methods

### Ethics Statement

Ethics approval was obtained from the University of Toronto Institutional Review Board and the study is registered with http://clinicaltrials.gov (NCT 01353014). All subjects provided written informed consent. The protocol for this trial and supporting CONSORT checklist are available as supporting information; see Checklist S1 and Protocol S1.

### Study Design and Subjects

**Figure 1:**
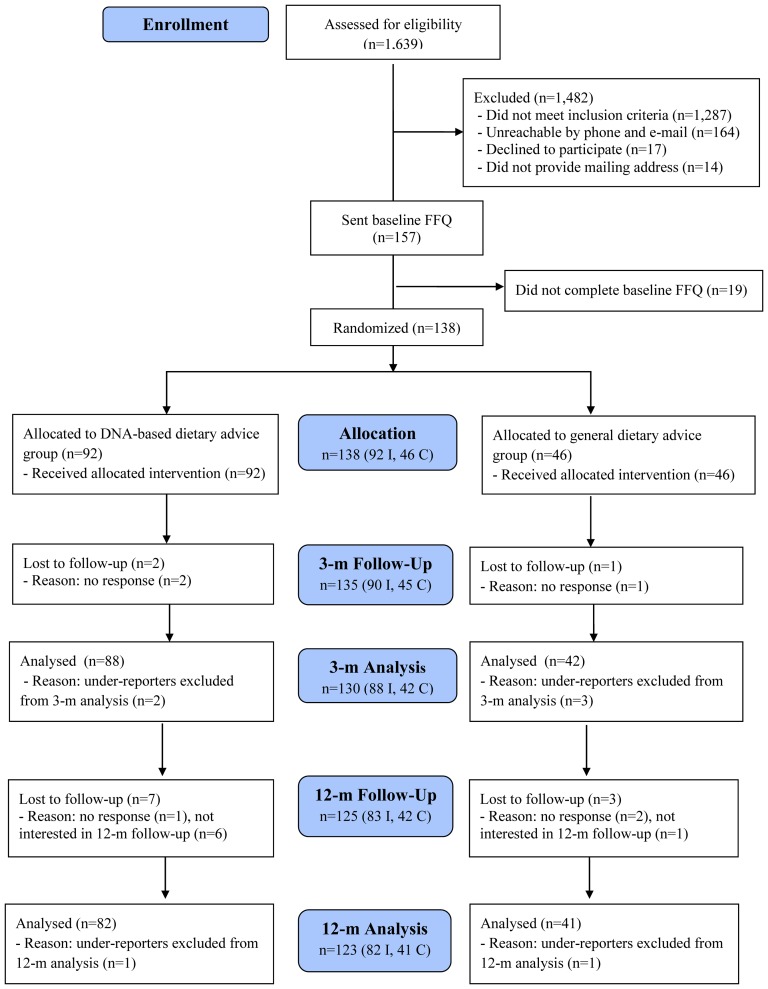
Consolidated standards of reporting trials (CONSORT) diagram and subject flow through the trial.

The present study was intended to mimic the nature of a direct-to-consumer genetic test such that all study materials were distributed and completed in the mail or electronically and no in-person contact was made with subjects for the present study. Details on the study design have been published elsewhere [13]. Briefly, subjects (n = 157) who had previously participated in a nutrigenomics research study and had provided a blood sample were invited to complete a 196-item, semi-quantitative Toronto-modified Willet food frequency questionnaire (FFQ) and were then randomized to an intervention or control group (Figure 1). Subject recruitment occurred from May 2011 to August 2011, the 3-month follow-up assessment occurred from September 2011 to January 2012 and the 12-month follow-up assessment took place from June 2012-October 2012. Since the recommendations in this study were based on caffeine, vitamin C, sugar, and sodium, eligible participants were those who consumed at least 100 mg of caffeine per day, 10% of energy from total sugars per day, and 1,500 mg of sodium per day and did not take vitamin C-containing supplements. Eligible women who were pregnant or breast-feeding at the time of recruitment were excluded from the study Subjects were given information on portion sizes, which were indicated for most FFQ items, and were asked to select how frequently they consumed the items over the past month from a list of frequency responses. The FFQ was used to collect detailed information on intake of fruits and vegetables, dairy products, meats and alternatives, grain products, sweets and baked goods, processed and prepared foods, and caffeinated and non-caffeinated beverages. Nutrient analyses were carried out at the Harvard School of Public Health Channing Laboratory using the USDA National Nutrient Database for Standard Reference.

Randomization was done by study personnel who used a computer software program (Random Allocation Software) that generated a random list of assignments. A 2∶1 ratio of participants in the intervention group compared to the control group was applied since the intervention group consisted of those who would have either the “risk” or “non-risk” genotype for each of the four genes (Table 1). Participants were informed that they would receive DNA-based dietary advice at some point during the study and those who were randomized to the control group were given the DNA-based advice after the final follow-up assessment was completed. Dietary intakes were self-reported by participants on the FFQ with no assistance from study personnel, and the nutrient analyses were made without knowledge of study group assignment. DNA was isolated from whole blood with the GenomicPrep Blood DNA Isolation kit (Amersham Pharmacia Biotech Inc, Piscataway, NJ) and genotyping was completed using either real-time polymerase chain reaction on an ABI 7000 Sequence Detection System (Applied Biosystems) or a multiplex restriction fragment length polymorphism (RFLP) polymerase chain reaction method, as described previously [16], [17]. Genotyping was verified by using positive control subjects in each 96-well plate as well as a second genotyping of ∼5% of a random selection of samples with 100% concordance.

### Dietary Reports and Recommendations

Subjects in the intervention group were genotyped for variants that affect caffeine metabolism (*CYP1A2*) [18], [19], vitamin C utilization (*GSTT1* and *GSTM1*) [17], sweet taste perception (*TAS1R2*) [20], and sodium-sensitivity (*ACE*) [21], [22] (Table 1). These genes were selected as representative sample tests from consumer genetic testing companies, which are based on studies of gene-diet interactions using a candidate gene approach. Although newer experimental approaches such as genome-wide association studies (GWAS) are being used to identify genetic variants associated with disease susceptibility, to date only a few GWAS studies have investigated gene-diet interactions on health outcomes [23], [24]. Thus, the purpose of the present study was to evaluate the behavioral response to disclosing genetic information, not to validate the clinical efficacy of the genetic variants provided in the dietary advice reports. Dietary reports were sent to all subjects by e-mail. The dietary report for subjects in the intervention group informed subjects of their genotypes and included a corresponding DNA-based dietary recommendation for daily intake of caffeine, vitamin C, added sugars and sodium (Nutrigenomix Inc., Toronto, Canada). Those who possessed the genotype that has been associated with increased risk of a health outcome when consuming above or below a certain daily amount were given a “targeted” dietary recommendation. For caffeine and sodium, this recommendation was more stringent than the current general recommendation for daily intake and was based on previous work that evaluated health outcomes according to genotype at different levels of intake [18], [19], [21], [22]. For added sugars and vitamin C, subjects were informed to be particularly mindful of meeting the current general recommendation for daily intake, since no previous study has examined how individuals respond to consuming various levels of these nutrients according to genotype and, therefore, a different intake level could not be recommended. Subjects who possessed the genotype that has not been associated with increased risk received the current general recommendation for daily intake [25]–[27]. The control group was e-mailed a report of current general recommendations for the same nutrients without genetic information. Subjects were e-mailed a monthly reminder of their dietary report and additional FFQs were collected at 3- and 12-month follow-up assessments.

### Study outcomes

The primary study outcome was change in dietary intakes of caffeine, vitamin C, added sugars, and sodium between the control and intervention groups from baseline to the follow-up assessments. Changes in dietary intakes were examined between baseline and 3-months to determine the short-term effects of the intervention, while changes in intakes between baseline and 12-months were examined to determine the long-term effects. The secondary study outcome was to compare the proportion of participants who met the recommendations for intake before and after the intervention for dietary components that significantly changed between the control and intervention groups.

### Statistical Analyses

Statistical analyses were performed using the Statistical Analysis Software (version 9.2; SAS Institute Inc., Cary, NC). The α error was set at 0.05 and all reported p-values are two-sided. Subject characteristics between the intervention and control group were compared using a Chi-square test for categorical variables and a Student's t-test for continuous variables. The distributions of nutrient intakes were examined and a log transformation was applied to those that deviated from normality. In these cases, the p-values from models using transformed values are reported, but untransformed means and measures of spread are reported to facilitate interpretation. Subjects who were likely under-reporters (consuming less than 800 kcal/day) were excluded from the analyses, since dietary intake data from these individuals may not have been reliable. Baseline mean intakes of vitamin C, sugar, sodium and caffeine were compared between ethnocultural groups using general linear models to determine if any significant dietary differences were present between groups at the start of the study. General linear models were also conducted to test for changes in dietary intakes between baseline and 3-months, and baseline and 12-months, in order to determine the effect of the dietary advice over a short- and long-term period. The Tukey-Kramer test for multiple comparisons was applied to determine whether any changes in intake of the intervention groups differed from the change in intake of the control group. The Chi-square test was used to compare the proportion of subjects meeting the recommendations for intake between baseline and the follow-up assessments. Fisher's Exact Test was used if a proportion category consisted of fewer than 5 subjects.

## Results

### Subject Characteristics

Of the 157 subjects who were sent the baseline FFQ, 125 completed the 12-month study giving an overall retention rate of 80% (Figure 1). In relation to those who were randomized (n = 138), this represents 91% of subjects who completed the 12-month study. The mean age of the participants was 26.5±3.0 years and 78% were female. The study population was multi-ethnic with Caucasian, East Asian, and South Asian groups representing the majority of ethnic backgrounds. Over half of the population possessed at least an undergraduate degree. There were no significant differences between the characteristics of participants in the intervention group when compared to the control group (Table 2). However, a significant difference in baseline sodium intake (mg/day) was observed between the East Asian and Caucasian groups (1837±147 vs. 2319±88, p = 0.03). As a result, the general linear models examining changes in dietary intakes are adjusted for ethnocultural group. At baseline, the proportion of subjects who did not meet the general recommendation for caffeine, vitamin C, added sugars and sodium were 9%, 14%, 24% and 39%, respectively. Thirty eight percent of subjects did not meet the targeted recommendation (for those with elevated risk) for caffeine intake at baseline, while 80% did not meet the targeted recommendation for sodium intake. The targeted recommendation for vitamin C and added sugars was the same as the general recommendations.

**Table 2:** Participant characteristics.

	Intervention(n = 92)	Control(n = 46)	p-value
n (%)			
Age (years)*	27±3	26±3	0.82
Female	69 (75)	37 (80)	0.48
Ethnicity			
Caucasian	59 (64)	24 (52)	0.18
East Asian	19 (21)	12 (26)	0.47
South Asian	9 (10)	6 (13)	0.56
Other	5 (5)	4 (9)	0.46
Education			
Some college or undergraduate training	9 (10)	8 (17)	0.20
College or undergraduate degree	50 (54)	22 (48)	0.47
Graduate degree	33 (36)	16 (35)	0.90

^*^Values shown are mean ± standard deviation.

### Changes in Dietary Intakes

Of the 138 subjects who were randomized, 135 completed the 3-month follow-up and 130 were included in the 3-month analyses (n = 5 under-reporters excluded). At 12 months, 125 subjects completed the follow-up and 123 were included in the analyses (n = 2 under-reporters excluded). There were no differences in the baseline characteristics (e.g. age, proportion of males/females) between those who were included in the final analysis and those who were not. Compared to the control group, no significant changes from baseline were observed for intakes of caffeine, vitamin C, added sugars, or sodium at the 3-month follow-up among subjects in the intervention group who carried a risk version of the corresponding gene (intervention risk group) or among subjects who carried the non-risk version (intervention non-risk group). At the 12-month follow-up, subjects in the intervention group who were informed that they possessed the risk version of the *ACE* gene, and who were given the targeted advice to consume below the Adequate Intake (AI) of 1500 mg/day of sodium, significantly reduced their mean sodium intake (mg/day) from baseline when compared to the control group (−287.3±114.1 vs. 129.8±118.2, p = 0.008), which did not receive genetic information and was given the general recommendation for sodium intake (Tolerable Upper Intake Level (UL): ≤2300 mg/day). The mean change in sodium intake (mg/day) among subjects who were informed that they possessed the non-risk version of the *ACE* gene, and who were advised to follow the general recommendation for sodium intake, did not differ from the change in intake of the control group at 12-months (−244.2±150.2 vs. 129.8±118.2, p = 0.11). The mean changes in intakes from baseline for caffeine, vitamin C and added sugars did not differ from the control group at the 12-month follow-up among either the intervention-risk or intervention non-risk groups (Table 3).

**Table 3:** Changes in dietary intake after 3-months and 12-months.

	Baseline (n = 133)			3-months (n = 130)			12-months (n = 123)		
	n	Mean ± SEM	p-value‡ compared to control group	n	Mean change ± SEM	p-value‡ compared to control group	n	Mean change ± SEM	p-value‡ compared to control group
Caffeine (mg/day)									
Intervention risk	46	181.4±16.8	0.82	45	–3.0±14.8	0.61	41	–18.9±18.8	0.92
Intervention non-risk	44	194.8±17.8	0.92	43	–24.7±15.3	0.66	41	1.5±19.4	0.99
Control	43	183.5±16.3		42	–7.3±14.8		41	–0.3±17.8	
Vitamin C (mg/day)									
Intervention risk	50	197.3±33.6	0.85	49	49.5±37.6	0.99	45	36.6±43.1	0.73
Intervention non-risk	40	226.2±35.1	0.96	39	–13.9±38.4	0.22	37	–58.4±43.5	0.42
Control	43	220.0±31.9		42	44.1±37.1		41	–21.4±40.1	
Added sugars (%e/day)									
Intervention risk	38	8.9±0.8	0.99	37	–0.9±0.9	0.18	33	0.4±0.9	0.98
Intervention non-risk	52	8.3±0.7	0.54	51	0.5±0.8	0.99	49	–0.4±0.8	0.85
Control	43	9.3±0.7		42	0.6±0.8		41	–0.4±0.8	
Sodium (mg/day)									
Intervention risk	63	2144.5±124.4	0.51	62	–143.0±109.0	0.20	56	–287.3±114.1	0.008
Intervention non-risk	27	2224.9±171.0	0.31	26	97.8±145.6	0.97	26	–244.2±150.2	0.11
Control	43	2000.8±131.2		42	82.2±119.2		41	129.8±118.2	

‡ p-values are for log-transformed values.

Results are adjusted for ethnicity.

At the 12-month follow-up assessment 66% of subjects in the intervention risk group, 65% of subjects in the intervention non-risk group and 68% of subjects in the control group met the general recommendation for sodium intake of ≤2300 mg/day. In addition, 34% of subjects in the intervention risk group, 19% of subjects in the intervention non-risk group and 24% of subjects in the control group met the targeted recommendation for sodium intake of ≤1500 mg/day. These proportions were not significantly different between the control and intervention groups. Among those in the intervention group who had the risk version of the *ACE* gene, 19% met the targeted recommendation of ≤1500 mg/day at baseline compared to 34% after 12 months (p = 0.06), and 59% met the general recommendation of ≤2300 mg/day at baseline compared to 66% after 12 months (p = 0.41).

## Discussion

The present study is the first to evaluate the effects of disclosing genetic information related to personalized nutrition on dietary intake and the findings show that DNA-based dietary advice results in greater changes in intake for some dietary components compared to population-based dietary advice. Dietary modification is an important health behavior for chronic disease prevention. Changes in health behaviors have not been frequently reported in previous studies that have investigated the effect of disclosing genetic information related to disease risk [28]–[31] and a 2010 Cochrane review concluded that disclosing genetic risk information for disease has little impact on actual behavior, although it has a small effect on one's intention to change [32]. However, the genomic information provided in those studies was related to disease susceptibility, not personalized nutrition, and the studies lacked a long-term follow-up assessment. Moreover, participants in previous studies were not provided with personalized recommendations on what behavioral strategies should be followed to mitigate disease risk. Results from the present study provide evidence that genetic testing for personalized nutrition may be more clinically useful for motivating favorable dietary changes than testing for disease susceptibility, since a change in sodium intake was observed after 12-months among the intervention risk group. In line with this finding, a previous study comparing a personalized, DNA-based weight loss diet with a traditional weight loss diet reported that subjects on the personalized diet had greater dietary adherence, longer-term maintenance of weight loss and greater improvements in fasting blood glucose levels [33]. In addition, a study investigating health behavior changes after revealing genetic risk for Alzheimer's disease reported that the addition of a vitamin E supplement was the most common change to vitamin or medication use among subjects who were informed that they were at greater genetic risk [34].

Although changes were observed in dietary intakes of sodium among the intervention-risk group of subjects at the 12-month follow-up, no changes in intakes of caffeine, vitamin C or added sugars were observed at either follow-up assessment. This may be due to the baseline intakes of these nutrients that were already mostly in line with the recommendations that were given to the subjects who possessed a risk allele, which is a limitation of the present study. Nevertheless, variants in other genes involved in reward pathways may play a role in one's ability to reduce consumption of some of these dietary components [16], [35]. Indeed, the National Human Genome Research Institute has recommended investigation into the potential for genomic information to improve behavior change interventions by customizing interventions to individuals based on genetic markers of adherence [36], [37]. Despite the lack of an intervention effect on intake of these three dietary components in the present study, it is worth noting that subjects who possessed a non-risk allele for the corresponding genes did not shift to a less desirable level of intake by increasing their consumption of caffeine, sodium or added sugars, or decreasing their intake of vitamin C. Although we did not report a detrimental impact on dietary intake behavior as a result of disclosing genetic information indicating no increased risk, proper communication of genetic test results is needed to prevent individuals from misunderstanding or misinterpreting the information and to guide them toward making appropriate lifestyle changes where necessary [38]. As such, providing this type of information through a qualified healthcare professional might be more appropriate than providing such information direct-to-consumer. If the results of a genetic test require dietary modification then a dietitian might be best suited to guide the consumer whereas a genetic counselor would be better suited for communicating results of tests for high penetrance genes that may require a more severe intervention.

Another limitation of the present study is the use of a FFQ to assess dietary intake, which is more useful in larger, population-based studies, as it provides a measure of relative intake rather than actual intake. However, the objective of the present study was to assess change in dietary intakes, which is a relative measure of intake. In addition, the sample size was small, yet comparable to previous studies examining the impact of disclosing genetic information on particular health behaviors [30], [33], [34], and subjects were highly educated and recruited from a previous nutrigenomics study. The reported reduction of nearly 300 mg of sodium per day in the intervention risk group was not sufficient to reduce the average sodium intake to the AI of 1500 mg/day, which was the targeted recommendation provided in the dietary report. Nevertheless, a recent Institute of Medicine report concluded that there is no benefit to sharply restricting sodium intake to the level of the AI [39] and a 2010 computer-simulated model examining the effect of dietary salt reduction on future cardiovascular disease projected that a 1 g/day reduction in average population salt intake, which is equivalent to about 400 mg/day of sodium, would prevent up to 28,000 deaths from any cause and would be more cost-effective than using medications to manage hypertension [40]. Therefore, the approximate 300 mg/day reduction in sodium intake reported in the present study would be considered clinically relevant.

Strengths of the present study are the inclusion of a control group, which provided a method of comparing the utility of DNA-based dietary advice to population-based recommendations, and the randomized design, which minimizes the potential for confounding effects. Including a 3- and 12-month follow-up assessment enabled us to examine the short- and long-term effects of the intervention. The finding that sodium intake was significantly reduced compared to the control group after 12-months among subjects in the intervention group with the risk version of the *ACE* gene suggests that longer-term studies are required to fully determine the impact of disclosing genetic information. Moreover, conducting the present study so that it closely resembles a consumer genetic test increases the validity of the findings to reflect the real world effects among consumer genetic test users. Early adopters of consumer genetic testing are more likely to be highly educated and Caucasian, with a substantial proportion of users between the ages of 18–49 years [15], [28], [41]. One study has reported a larger proportion of female consumers [41]. As a result, the subjects in the present study are representative of the early adopters of consumer genetic testing.

The present study was the first to empirically test the effect of DNA-based personalized nutritional advice on dietary intake behavior compared to population-based dietary advice. The findings show that DNA-based dietary advice can impact dietary intake to a greater extent than general population-based recommendations and provide supportive evidence for the clinical utility of personalized nutrition to assist in chronic disease prevention.

## Supporting Information

Checklist S1 **CONSORT Checklist.**
(DOC)

Protocol S1 **Trial Protocol.**
(DOCX)
