# Supplementary material for: Disclosure of Genetic Information and Change in Dietary Intake: A Randomized Controlled Trial
Source: PLoS One. 2014 Nov 14;9(11):e112665. doi: 10.1371/journal.pone.0112665 (PMC4232422; doi:10.1371/journal.pone.0112665)
Supplement: Protocol S1 — Trial Protocol. (DOCX) [file pone.0112665.s002.docx]

**Trial Protocol and Statistical Analysis Plan**

Title: Disclosure of genetic information and change in dietary intake: a randomized

controlled trial

Authors: Daiva E Nielsen and Ahmed El-Sohemy

This supplement contains the following items:

1. Summary of trial
2. Original trial protocol, final protocol, summary of changes
3. Original statistical analysis plan, final statistical analysis plan, summary of changes
4. **Summary of trial**

The purpose of this randomized controlled trial is to determine the effect of disclosing diet-related genetic information on dietary intake behaviour. Participants who completed the Toronto Nutrigenomics and Health study will be invited by e-mail to participate in this study. Informed consent will be obtained. Participants who choose to take part will be randomized into an intervention group or a control group (2:1 ratio). All participants will complete a baseline196-item food frequency questionnaire (FFQ) to assess habitual food intake over the past 30 days. Shortly after the baseline assessment, participants in the intervention group will receive a report of personalized dietary recommendations. The report will provide them with information on their gene variants that influence caffeine metabolism, sugar consumption, salt sensitivity and vitamin C metabolism. Participants provided a blood sample for the Toronto Nutrigenomics and these genotypes were determined. The report will explain what the genotypes mean and will provide DNA-based advice for daily intakes of caffeine, sugar, sodium and vitamin C. Participants in the control group will receive Health Canada’s general recommendations for intakes of caffeine, sugar, sodium and vitamin C with no genetic information. Three months after receiving the dietary recommendations, participants in both groups will complete a second FFQ to examine potential short term changes in dietary intake. A third FFQ will be collected one year after baseline, to examine potential long term changes. The nutrient analysis between the three FFQs will be compared to identify changes in dietary intake of caffeine, sugar, sodium and vitamin C. The intervention group FFQs will be compared to the control group FFQs to examine how changes differed when dietary advice was personalized based on genotype.

**Significance:** Recent advances in genomics technology have made the acquisition of personalized genetic information easily obtainable. Nutrigenomics tests offering dietary advice based on genotype are commercially available through the internet (1). A potential outcome of these genetic tests is better dietary and lifestyle habits for improved health. However, much debate exists about the influence of genetic information on lifestyle modification (2). We are pursuing research in this area to create new knowledge of how providing genetic information affects individual dietary intake behaviour.

**References:**

1. Sterling R (2008) The online promotion and sale of nutrigenomic services. Genet Med. 10:784-796.

2. McBride CM, Koehly LM, Sanderson SC, Kaphingst KA (2010) The behavioral response to personalized genetic information: will genetic risk profiles motivate individuals and families to choose more healthful behaviors? Annu Rev Public Health. 31:89-103.

1. **Original trial protocol**

- 200 subjects (aged 20-35 years) will be recruited from the Toronto Nutrigenomics and

Health Study.

- Exclusion criteria:

o <100 mg of caffeine/day

o <10% energy from total sugars/day

o <1500 mg of sodium/day

o Vitamin C supplement use

o Pregnant/nursing

- Subject characteristics (age, sex, ethnicity, level of education) will be collected using a

questionnaire.

- A food frequency questionnaire (FFQ) will be collected at 0, 3 and 12 months.
- Subjects will be randomized to an intervention group or control group (2:1 ratio) and

dietary advice reports will be given.

- Control group: general dietary recommendations for caffeine, vitamin C, added

sugars and sodium.

- Intervention group: DNA-based dietary recommendations for caffeine, vitamin C,

added sugars and sodium.

- Copy of dietary advice reports given at monthly intervals.

**Final protocol:**

Same as original protocol except 157 subjects were recruited.

S**ummary of changes:**

No changes were made to the original study protocol. 157 subjects were recruited instead of the

original goal of 200. Routine updates were made to the protocol’s trial record on

http://clinicaltrials.gov (NCT01353014) to reflect study progress (e.g. enrolment status,

anticipated follow-up and completion dates, actual follow-up and completion dates).

1. **Original statistical analysis plan**

- Subject characteristics between the intervention and control group will be compared using

a Student’s t-test for continuous variables (age) and a Chi-square test for categorical

variables (sex, ethnicity, level of education).

- General linear model will be used to identify changes in the mean intakes of caffeine,

added sugars, vitamin C and sodium from baseline to 3 months and baseline to 12 months.

- Tukey’s test for multiple comparisons will be applied to determine whether changes in

intake of the intervention risk and intervention non-risk groups differ from the change in

intake of the control group.

**Final statistical analysis plan (changes from original plan are indicated in bold):**

- Subject characteristics between the intervention and control group will be compared using

a Student’s t-test for continuous variables (age) and a Chi-square test for categorical

variables (sex, ethnicity, level of education).

- General linear model used to identify changes in the mean intakes of caffeine, added

sugars, vitamin C and sodium from baseline to 3-months and baseline to 12-months.

- Tukey’s test for multiple comparisons applied to determine whether changes in intake of

the intervention risk and intervention non-risk groups differed from the change in intake of

the control group.

- **General linear model used to compare baseline intakes of caffeine, vitamin C, added**

**sugars and sodium between ethnic groups (Caucasian, East Asian and South Asian).**

- **Ethnicity was adjusted for in dietary intake analyses if a significant difference was**

**observed at baseline between ethnic groups.**

**Summary of changes:**

- General linear model used to compare baseline intakes of caffeine, vitamin C, added sugars

and sodium between ethnic groups (Caucasian, East Asian and South Asian). Ethnicity was

adjusted for in dietary intake analyses if a significant difference was observed at baseline

between ethnic groups.

- - **Rationale:** A significant difference in baseline nutrient intake was observed

between two ethnic groups. Therefore, we modified our statistical analysis plan to

include ethnicity as a covariate in the general linear model used to identify

changes in nutrient intakes.
